# Risk factors for heart failure in a cohort of patients with newly diagnosed myocardial infarction: a matched, case-control study in Iran

**DOI:** 10.4178/epih.e2016019

**Published:** 2016-05-17

**Authors:** Ali Ahmadi, Koorosh Etemad, Arsalan Khaledifar

**Affiliations:** 1 Department of Epidemiology and Biostatistics, Modeling in Health Research Center, Shahrekord University of Medical Sciences, Shahrekord, Iran; 2 Department of Epidemiology, School of Public Health, Shahid Beheshti University of Medical Sciences, Tehran, Iran; 3 Department of Cardiology, School of Medicine, Hajar Hospital, Modeling in Health Research Center, Shahrekord University of Medical Sciences, Shahrekord, Iran

**Keywords:** Heart failure, Epidemiology, Myocardial infarction, Mortality, Risk factors, Case-control studies

## Abstract

**OBJECTIVES:**

Risk factors for heart failure (HF) have not yet been studied in myocardial infarction (MI) patients in Iran. This study was conducted to determine these risk factors.

**METHODS:**

In this nationwide, hospital-based, case-control study, the participants were all new MI patients hospitalized from April 2012 to March 2013 in Iran. The data on 1,691 new cases with HF (enrolled by census sampling) were compared with the data of 6,764 patients without HF as controls. We randomly selected four controls per one case, matched on the date at MI and HF diagnosis, according to incidence density sampling. Using conditional logistic regression models, odds ratios (ORs) with a 95% confidence interval (CI) were calculated to identify potential risk factors.

**RESULTS:**

The one-year in-hospital mortality rate was 18.2% in the cases and higher than in the controls (12.1%) (p<0.05). Significant risk factors for HF were: right bundle branch block (RBBB) (OR, 2.86; 95% CI, 1.95 to 4.19), stroke (OR, 2.00; 95% CI, 1.39 to 2.89), and coronary artery bypass grafting (CABG) (OR, 2.03; 95% CI, 1.34 to 3.09). Diabetes, hypertension, percutaneous coronary intervention (PCI), atrial fibrillation, ventricular tachycardia, and age were determined to be the factors significantly associated with HF incidence (p<0.05). The most important factor in women was diabetes (OR, 1.41; 95% CI, 1.05 to 1.88). Age, hypertension, PCI, CABG, and RBBB were the most important factors in men.

**CONCLUSIONS:**

Our findings may help to better identify and monitor the predictive risk factors for HF in MI patients. The pattern of risk factors was different in men and women.

## INTRODUCTION

Heart failure (HF) is a serious challenge facing public health and health care systems. HF is a condition in which the heart cannot pump adequate blood for the body’s metabolic needs. HF is a clinical syndrome that occurs in patients with a congenital and/or acquired defect in heart structure or function, and leads to repeated hospitalizations and decreased life expectancy. Worldwide, HF is the cause of many deaths [1-4].

HF is the outcome of many cardiovascular diseases, including myocardial infarction (MI) and its associated risk factors such as hypertension, diabetes, and chronic renal disease. Mortality due to HF remains worryingly high despite improvements in treatment [5].

Epidemiological studies have indicated that HF leads to a noticeable decline in quality of life [6,7]. Despite improvement and promotion of approaches to treat cardiovascular disease, HF remains an important health problem. Assessment and monitoring of mortality, and its associated factors, are considered vital to HF health care. Management, prevention, and control of HF require its precise detection, with an epidemiological approach in communities and throughout the country [3-5,8]. The epidemiology of HF has been well explained, with gradual updates for developed nations [2-4], and it has been reported for some Asian countries like Japan, India, and Malaysia [8,9]. However, no comparison of risk factors for HF has yet been made in cardiac disease patients with or without HF worldwide, particularly between men and women. HF epidemiology has not yet been studied in Iran, one of the largest Middle Eastern countries, in Southwest Asia. Cardiovascular diseases, especially MI, are the leading cause of mortality in Iran and are currently being addressed as the most important challenge facing Iran’s health care system [10,11]. This study was conducted to determine the risk factors for HF in Iranian MI patients.

## MATERIALS AND METHODS

This study is a hospital-based, nested, case-control study. The study protocol was approved by an independent scientific review committee (no. 1392-1-85-12129). The cohort of participants consisted of all new MI patients hospitalized from April 2012 to March 2013 in hospitals with a cardiology ward in Iran. In this study, the data of 20,750 MI patients in 540 hospitals in Iran were analysed. The cohort of the study consisted of patients registered in the Iranian Myocardial Infarction Registry of the Iran Health and Medical Education Ministry (Department of Cardiovascular Disease Prevention) in hospitals with a cardiology ward across the 31 provinces of Iran using the date of arrival and date of discharge from the hospital. Data collection is mandatory in all hospitals, although some private and military hospitals do not participate. These data have already been used in other studies [10,11]. In this study, the data on 1,691 new cases with HF were compared with those of 6,764 patients without HF (the controls). The cases were selected by census throughout one year of study. The controls were selected, concurrently with the cases, from patients without HF during hospitalization due to MI.

The sampling method was random. The controls and cases were matched on the date of hospitalization and MI. We determined the date of death and also of MI and HF diagnosis. Then, to obtain an unbiased estimate of rate ratios, we randomly selected four controls per one case matched on the date at MI and HF diagnosis (according to calendar year) according to incidence density sampling (as per the original study; personal correspondence). HF and the associated in-hospital mortality were the main outcomes. According to the inclusion and exclusion criteria, this study obviously investigated new cases (rather than prevalence) of HF and in-hospital mortality in the patients. The diagnosis of HF was made based on the National Protocol of the Iran Ministry of Health and Medical Education (per the International Classification of Diseases, 10th revision) by a cardiologist. In Iran, the Framingham clinical criteria (the presence of two major criteria or one major criterion plus two minor criteria) and an echocardiogram are used to diagnose HF. Clinical examination of the Framingham criteria are not accepted as sufficient for diagnosing HF alone, as symptoms may be non-specific and hence physical findings may not have adequate sensitivity for an accurate diagnosis. Therefore, these criteria are more valuable when accompanied by an echocardiogram. The major criteria consist of paroxysmal nocturnal dyspnea, neck vein distention, rales, radiographic cardiomegaly, acute pulmonary edema, S_3_ gallop, increased central venous pressure (>16 cm H_2_O at right atrium), hepatojugular reflux, and weight loss of >4.5 kg in five days in response to treatment, and the minor criteria are bilateral ankle edema, nocturnal cough, dyspnea on ordinary exertion, hepatomegaly, pleural effusion, a decrease in vital capacity by one third from maximum recorded, and tachycardia (heart rate >120 beats/ min). The patients underwent echocardiogram as soon as possible (at latest up to the next morning after referral to the hospital). Echocardiography was done per Simpson’s biplane and tissue Doppler. Patients with none of the Framingham diagnostic criteria and no definite diagnosis of HF by echocardiography were excluded from the study. The covariates included in the analyses were age, gender, literacy, smoking, body mass index (BMI), the season at disease incidence, and the comorbidities (type 2 diabetes, hypertension, hypercholesterolemia, and congenital heart disease). The above covariates were registered and gathered as part of the patient’s medical record, at definite diagnosis of the disease. A cardiologist diagnosed MI based on the data on left bundle branch block (LBBB), right bundle branch block (RBBB), atrial fibrillation (AF), and ventricular tachycardia (VT) with reference to Braunwald’s heart disease [12] (for example, common diagnostic criteria for LBBB: QRS duration ≥120 ms broad, notched, or slurred R waves in leads I, aVL, V_5_, and V_6_, small or absent initial r waves in right precordial leads (V_1_ and V_2_) followed by deep S waves, absent septal q waves in leads I, V_5_, and V_6_, prolonged time to peak R wave (>60 ms) in V_5_ and V_6_; for RBBB: QRS duration ≥120 ms, rsr’, rsR’, or rSR’, patterns in leads V_1_ and V_2_, S waves in leads I and V_6_ ≥40 ms wide normal time to peak R wave in leads V_5_ and V_6_ but > 50 ms in V1) [10-12].

### Statistical analysis

The MI patients with HF as the cases were compared with a group of the MI patients without HF as the control group. The odds ratio (OR) with a 95% confidence interval (CI) of the factors associated with incidence of HF was calculated by conditional logistic regression.

The best statistical model was selected using Akaike information criterion and McFadden’s pseudo R^2^. In the regression analysis, first, univariate analysis was conducted. Then, the variables with a significance value of approximately p=0.2 were entered into the multivariate model to adjust for potential confounding variables. In univariate analysis, gender was not significant for HF incidence. When analysis was performed according to gender, the model’s coefficients and the significant values of the HF risk factors were different. Therefore, the final modelling was done for men, women, and all the patients separately. To enter quantitative variables such as age into regression modelling, the variables were standardized. The p-values less than 0.05 were considered significant. Data analysis was done using Stata version 13.0 (StataCorp, College Station, TX, USA).

## RESULTS

Overall, 6,131 (72.5%) patients were men and 2,324 (27.5%) were women. Of 1,691 cases, 497 (29.4%) were women and 1,194 (70.6%) were men, and of 6,764 controls, 1,827 (27.0%) were women and 4,937 (73.0%) were men. The mean±standard deviation (SD) age at HF diagnosis was significantly higher in the case group than the controls. The mean±SD duration of hospitalization was 6.5±14.6 days in all patients.

The prevalence of smoking, hypertension, and diabetes was significantly higher in the case group than the controls (p<0.05). The descriptive data of all patients and comparison between the cases and the controls are shown in Table 1.

Use of percutaneous coronary intervention (PCI) and coronary artery bypass grafting (CABG) was significantly higher in the case group than the control group (<0.001). Furthermore, the prevalence of RBBB, LBBB, AF, VT, and stroke was significantly higher in the case group than the controls (p<0.001). The comparison of clinical data between the cases and the controls is shown in Table 2.

As all the HF patients were enrolled in the study by census, the spatial and time distribution of the HF patients in Iran was as follows: the highest prevalence of HF was obtained in Khuzestan province (9.0%), followed by Gilan (7.5%), and the lowest prevalence in Zanjan and in Kohgiluyeh and Boyer-Ahmad (0.5%). Regarding the time distribution, the highest prevalence rate was obtained in January and December (the cold season in Iran) (14.0% and 10.0%, respectively) and the lowest in July and June (the hot season in Iran) (5.6% and 5.9%, respectively).

Within one year of the study, the mortality rate (case fatality rate) in the case and control groups was determined to be 18.2% and 12.1%, respectively, with a significant difference (p<0.05).

In univariate analysis, gender was not significant for HF incidence. We performed a stratified analysis by gender with univariate and multiple analysis. The highest OR of HF in all the patients was obtained for RBBB (2.86; 95% CI, 1.95 to 4.19). The ORs yielded by a conditional logistic model to determine the factors associated with HF are shown in Tables 3 and 4. With an increase in age by one year, the likelihood of HF incidence increased by 1.13 times. Stroke incidence more than doubled the likelihood of HF incidence (OR, 2.00; 95% CI, 1.39 to 2.89). Age, type 2 diabetes, hypertension, PCI, CABG, LBBB, RBBB, AF, VT, and stroke were among the significant determining factors for HF incidence.

Table 4 shows that the pattern of significant and predictive risk factors for HF incidence is different in men and women. The signifcant risk factors for HF were hypertension (p=0.001) in men and diabetes (p=0.02) in women. Age was determined to be a significant risk factor in men. Stroke, age, LBBB, CABG, and PCI were not significant for HF incidence in women. The significant risk factors for HF were hypertension in men and diabetes in women.

## DISCUSSION

The findings of our study plotted the HF epidemiology (descriptive and analytical) using an appropriate and robust methodology, for the first time, at a national scale in Iran. Our study showed that the most important risk factors for HF in MI patients were type 2 diabetes, hypertension, stroke, RBBB, CABG, and age. In addition, our study reported the ORs of risk factors for HF in MI patients in Iran by gender and showed some differences in risk factors between men and women. HF is a common complication of MI. Different factors—including infarct size, ventricular remodeling, stunned myocardium, recurrent myocardial ischemia, mechanical complications, and hibernating myocardium—affect the development of left ventricular systolic dysfunction with or without clinical HF after MI [13]. In this study, HF was seen more in men than women and its prevalence greatly increased with age. This finding is consistent with the study by Minicucci et al. [14]. In epidemiological studies, the rate of signs and symptoms of HF after MI has been reported to be approximately 25%. Importantly, this finding may be consistent with several clinical trials as well as our study. Moreover, approximately 40% of MIs are associated with left ventricular systolic dysfunction [15].

Therefore, the available data show that HF after MI is very frequent. This event had a prevalence of 8.14% (1,691 HF cases in 20,750 MI patients) in our study in Iran. In the study conducted by Grazuleviciene & Dulskiene [16] in Kaunas, Lithuania from 1997 to 2000, 448 patients with first-time MI were interviewed and enrolled. The prevalence of HF for 25-year-old to 64-year-old men with first MI showed that the syndrome was found in 46.4% of patients. Our finding is inconsistent with this study.

In our study, one-year prevalence of HF was reported, but in the study by Grazuleviciene & Dulskiene [16], four-year prevalence of HF was reported. The inconsistency of the findings between these two studies may be related partly to differences in the time, the duration, and the populations of the two studies. The prevalence of hypertension was 50.5% in their study, whereas the prevalence was 42.7%, 58.9%, and 35.9%, in the total population, the women population, and the men population of our study, respectively. In their study, the OR of hypertension in MI patients, a risk factor for HF, was reported as 1.51, which is consistent with our study. Besides that, overweight and impaired glucose tolerance were reported to be risk factors. In our study, diabetes was introduced as a risk factor. In these respects, our study findings are in line with that of Grazuleviciene & Dulskiene [16] and other studies [17,18].

Health care for patients with HF in Iran is based mainly on the evidence obtained from clinical trials and works conducted in the US and Europe. No clinical trial, case-control study, or prospective cohort study has yet been conducted in Iran to identify the characteristics of HF in MI patients and to explain its epidemiology. The findings of this study could be an important reference for physicians and researchers. In our study, the HF mortality ratio in MI patients was reported to be 18.2%, higher than the 13% reported by the study of Velazquez et al. [17] and higher than was found in studies conducted on the general populations in some Asian countries such as Japan, China, and Malaysia (3.9% to 6.7%). According to the previous studies, the prevalence of HF varies in Asia (1.3% to 6.7%). Our study reports the highest prevalence of HF in MI patients among Asian countries to date [9,14,19-21].

In a study conducted in Alberta, Canada between 1994 and 2000, 7,733 patients aged 65 years and over with MI were followed up during hospital stay and 37% were reported to be diagnosed with HF. In these patients, the case fatality rate was reported to be 13%. Moreover, 37.8% of the patients were female, 23.2% had diabetes, 42.3% hypertension, and 20.4% AF. The fatality rate reported in our study was higher than in this study [22], but the prevalence of risk factors was approximately similar. The prevalence of clinical risk factors, including AF, was reported to be much lower in Iran than in the study by Ezekowitz et al. [22]. However, the difference in the ages of the populations studied, most probably the difference in approaches to diagnosis and misclassification, under-reporting, and lack of accurate diagnosis in Iran, where there is less extensive access to MI healthcare and high mortality due to MI, can explain the inconsistent findings concerning the prevalence of risk factors. For example, the prevalence of PCI and CABG was higher in the study by Ezekowitz et al. [22] than in our study.

High incidence of HF and the associated mortality in MI patients in Iran could be explained by the patients’ late referral to emergency and cardiac care units (a study in Iran reported that a delay of more than eight hours after the onset of heart pain occurred in 65.5% of cases), lack of diagnostic facilities across the country, and unequal access to cardiac care. In addition, another reason may be the lack of a common PCI therapeutic strategy, which may decrease in-hospital mortality in cardiovascular disease patients (only 6.9% of the patients received PCI) [23,24]. Most patients were 30 years to 64 years old in our study and over 65 years old in other studies. The difference in age-related incidence patterns between our study and other studies deserves further investigation. In our study, the prevalence of stroke history was 2.7% in the patients. In another study, previous stroke was reported at approximately 10% in HF patients, which is lower than in our study [25]. In our study, AF had a significant role in HF incidence in MI patients. This finding is in agreement with the findings of the Framingham Heart Study. The coincidence of HF and AF had a worse prognosis than the incidence of only HF or AF [26]. The determinacy of diabetes and comorbidities in our study is consistent with other studies [14,16,22] and with studies conducted in Italy and Western Australia [27,28].

Because of epidemiologic transition, the increased elderly population of Iran, and critically high prevalence risk factors for cardiovascular diseases in both men and women (only 4% in the 15-year-old to 44-year-old population and 1% in the 45-year-old to 64-year-old population had no cardiovascular disease risk factors) [23,24], a silent epidemic of HF as the final outcome of cardiovascular diseases seems to be underway, which has not yet been addressed. Our results can be used in evidence-based decision making, research, prioritization, and planning in health care systems, in order to obtain better knowledge of HF etiology in MI patients and its risk factors, as well as for assessing preventive approaches [29,30]. The strength of our study is an appropriate inclusion of all HF patients within one year by census in Iran, efficient methodology and consideration of individual, time, and place in descriptive epidemiology, as well as the factors relevant to HF in MI patients in analytical epidemiology. Besides that, a large, hospital-based sample size is a unique strength of this study, as the first national study in Iran. A lack of data gathered on the patients, no HF classification, and no similar works in Iran and neighbouring countries for comparison and determination of trends are the limitations of this study, which should be considered in future works.

In summary, to the best of our knowledge, we found no study that investigates the risk factors for HF in MI patients stratified by gender. Therefore, this may be considered the most remarkable strength of our study. In our study, the incidence pattern of HF and its associated factors in MI patients was different in men and women. Accordingly, the ORs of risk factors for HF in MI patients were different in men and women. The most important risk factors for HF were diabetes, RBBB, AF, and VT in women MI patients. In contrast, the most important risk factors for HF were age, hypertension, stroke, VT, AF, RBBB, LBBB, CABG, PCI, and chest pain resistant to treatment in men MI patients. Risk factors were more frequent in men than women. The highest OR of HF was obtained for RBBB in all MI patients.

## Figures and Tables

**Table 1.** Comparison of the demographic characteristics and medical history at baseline (the date at heart failure diagnosis) in cases and controls, adjusted for gender

Characteristics	Cases (n = 1,691)			Controls (n = 6,764)			p-value^1^
	Total	Men	Women	Total	Men	Women		
Age (mean±SD, yr)	63.7 ±13.4	61.9± 13.3	68.2±12.3	61.5±13.3	59.9±13.2	65.8±12.9	<0.001
Illiteracy	883 (52.2)	503 (42.1)	380 (76.4)	3,159 (46.7)	1,836 (37.1)	1,323 (72.4)	0.05
Smoking	722 (42.7)	337 (28.2)	89 (17.9)	2,399 (35.4)	1,395 (28.2)	333 (18.2)	0.001
Hypertension	722 (42.7)	429 (35.9)	293 (58.9)	2,399 (35.4)	1,419 (28.7)	980 (53.6)	<0.001
Diabetes	476 (28.2)	265 (22.1)	211 (42.4)	1,521 (22.5)	901 (18.2)	620 (33.9)	<0.001
Hypercholesterolemia	319 (18.8)	180 (15.0)	139 (27.1)	1,196 (17.6)	740 (14.9)	456 (24.9)	0.26
PCI	152 (8.9)	107 (8.9)	45 (9.0)	447 (6.6)	341 (6.9)	106 (5.8)	<0.001
CABG	82 (4.8)	61 (5.1)	21 (4.2)	186 (2.7)	148 (3.0)	38 (2.0)	<0.001

Values are presented as number (%). SD, standard deviation; PCI, percutaneous coronary intervention; CABG, coronary artery bypass grafting.

1 Comparison of cases with controls.

**Table 2.** Comparison of the clinical factors and co-morbidity at baseline (the date at heart failure diagnosis) in cases and controls, adjusted for gender

Characteristics	Cases (n = 1,691)			Controls (n = 6,764)			p-value^1^
	Total	Men	Women	Total	Men	Women	
LBBB	55 (3.2)	43 (3.6)	12 (2.4)	120 (1.7)	77 (1.5)	43 (2.3)	< 0.001
RBBB	53 (3.1)	40 (3.3)	13 (2.6)	62 (0.9)	47 (0.9)	15 (0.8)	< 0.001
AF	92 (5.5)	61 (5.1)	31 (6.2)	206 (3.1)	142 (2.8)	64 (3.5)	< 0.001
VT	137 (8.2)	97 (8.1)	40 (8.0)	331 (4.8)	234 (4.7)	97 (5.3)	< 0.001
Stroke	46 (2.7)	37 (3.1)	9 (1.8)	91 (1.3)	81 (1.6)	10 (0.5)	< 0.001
STEMI	1,008 (59.6)	728 (60.9)	280 (56.3)	4,331 (64.0)	3,203 (64.8)	1,128 (61.7)	0.001
Thrombolytic	656 (38.7)	480 (40.2)	176 (35.4)	1,614 (23.8)	2,247 (45.5)	740 (40.5)	< 0.001
Hospital mortality	308 (18.2)	196 (16.4)	112 (22.5)	821 (12.1)	553 (11.2)	268 (14.6)	< 0.001
Hospital stay	6.8±15.7	7.4±16.8	5.5±12.5	28.0±36.0	28.3±36.0	27.5±36.0	< 0.001

Values are presented as number (%). LBBB, left bundle branch block; RBBB, right bundle branch block; AF, atrial fibrillation; VT, ventricular tachycardia; STEMI, ST-segment elevation myocardial infarction.

1 Comparison of cases with controls.

**Table 3.** Unadjusted odds ratio for risk factors for heart failure in men and women myocardial infarction patients

Characteristics	Women	p-value	Men	p-value	Total	p-value
Age	1.61 (1.42, 1.83)	0.001	1.29 (1.19, 1.40)	0.001	1.40 (1.31, 1.50)	0.001
Illiteracy	1.29 (1.00, 1.68)	0.05	1.40 (1.20, 1.64)	0.001	1.47 (1.29, 1.67)	0.001
Smoking	1.13 (0.85, 1.49)	0.38	1.36 (1.15, 1.60)	0.001	1.24 (1.08, 1.43)	0.002
Hypercholesterolemia	1.24 (0.97, 1.58)	0.08	1.03 (0.83, 1.27)	0.78	1.17 (1.00, 1.37)	0.05
Diabetes	1.39 (1.04, 1.63)	0.02	1.37 (1.14, 1.64)	0.001	1.42 (1.23, 1.63)	0.001
Hypertension	1.37 (1.10. 1.72)	0.005	1.09 (0.92, 1.28)	0.30	1.26 (1.11, 1.43)	0.001
Stroke	5.81(2.34, 14.39)	0.001	6.69 (4.62, 9.68)	0.001	6.15 (4.37, 8.65)	0.01
AF	2.60 (1.67, 4.06)	0.001	1.10 (0.73, 1.67)	0.63	1.59 (1.19, 2.14)	0.002
VT	2.70 (1.85, 3.93)	0.001	2.27 (1.73, 2.96)	0.001	2.41 (1.94, 2.99)	0.001
LBBB	1.44 (0.75, 2.76)	0.27	6.48 (4.48, 9.36)	0.001	4.21 (3.08, 5.75)	0.001
RBBB	2.45 (1.10, 5.47)	0.03	3.31 (2.09, 5.25)	0.001	3.02 (2.02, 4.50)	0.001
CABG	1.94 (1.08, 3.48)	0.03	1.46 (1.01,2.12)	0.04	1.54 (1.13, 2.11)	0.006
PCI	0.30 (0.15, 0.60)	0.001	0.44 (0.29, 0.65)	0.001	0.39 (0.28, 0.55)	0.001
Thrombolytic	0.55 (0.44, 0.68)	0.001	0.46 (0.39, 0.53)	0.001	0.49 (0.43, 0.56)	0.001
Chest pain	5.71 (4.37, 7.44)	0.001	6.95 (5.79, 8.35)	0.001	6.57 (5.65, 7.65)	0.001

Values are presented as odds ratio (95% confidence interval). AF, atrial fibrillation; VT, ventricular tachycardia; LBBB, left bundle branch block; RBBB, right bundle branch block; CABG, coronary artery bypass grafting; PCI, percutaneous coronary intervention.

**Table 4.** Adjusted odds ratio for risk factors for heart failure in men and women myocardial infarction patients

Characteristics	Women	p-value	Men	p-value	Total	p-value
Age	1.10 (0.95, 1.28)	0.19	1.14 (1.06, 1.23)	0.001	1.13 (1.07, 1.20)	0.001
Diabetes	1.41 (1.05, 1.88)	0.02	1.14 (0.96, 1.35)	0.12	1.25 (1.10, 1.34)	0.001
Hypertension	1.08 (0.80, 1.45)	0.60	1.29 (1.12, 1.50)	0.001	1.20 (1.06, 1.34)	0.002
Stroke	6.77 (0.75, 6.80)	0.09	1.80 (1.16, 2.78)	0.008	2.00 (1.39, 2.89)	0.001
AF	2.39 (1.19, 4.82)	0.01	1.63 (1.16, 2.28)	0.004	1.64 (1.26, 2.12)	0.001
VT	2.06 (1.14, 3.72)	0.02	1.64 (1.25, 2.16)	0.001	1.73 (1.39, 2.14)	0.001
LBBB	1.46 (0.57, 3.71)	0.42	1.62 (1.05, 2.51)	0.03	1.53 (1.09, 2.15)	0.01
RBBB	3.25 (1.01, 10.39)	0.05	3.26 (2.00, 5.33)	0.001	2.86 (1.95, 4.19)	0.001
CABG	0.87 (0.28, 2.65)	0.81	2.38 (1.39, 4.09)	0.002	2.03 (1.34, 3.09)	0.001
PCI	1.23 (0.73, 2.05)	0.43	1.39 (1.08, 1.80)	0.01	1.45 (1.19, 1.77)	0.001
Thrombolytic	0.76 (0.56, 1.03)	0.09	0.85 (0.74, 0.98)	0.03	0.83 (0.74, 0.93)	0.002
Chest pain	1.16 (0.75, 1.77)	0.49	1.32 (1.07, 1.65)	0.01	1.38 (1.17, 1.63)	0.001

Values are presented as odds ratio (95% confidence interval). AF, atrial fibrillation; VT, ventricular tachycardia; LBBB, left bundle branch block; RBBB, right bundle branch block; CABG, coronary artery bypass grafting; PCI, percutaneous coronary intervention.
